# Supplementary material for: LPD-YOLOv7-tiny: An enhanced lightweight YOLOv7-tiny model for real-time potato quality detection
Source: PLoS One. 2025 Oct 23;20(10):e0332043. doi: 10.1371/journal.pone.0332043 (PMC12548852; doi:10.1371/journal.pone.0332043)
Supplement: Attachment — Submitted filename: Responses to editor.docx [file pone.0332043.s003.docx]

1. The format of the manuscript has been revised as required.
2. Part of the data set in the article comes from open Internet resources, and part comes from the team's independent acquisition. The collection of data is in accordance with the terms and conditions of the data source.
3. The models and modules used in this paper are all open source resources, which can be widely obtained on the Internet.
4. "The funders had no role in study design, data collection and analysis, decision to publish, or preparation of the manuscript" has been added to the manuscript.
5. Data is contained within the article or supplementary material. All relevant data are on Figshare: https://doi.org/10.6084/m9.figshare.29458949
6. OrcID ID has been added.
7. We added a statement on the image data in the methods section of the article, stating that the collection and analysis methods comply with the terms and conditions of the data sources. Specifically:

Note: Some of the images in this study are derived from publicly accessible pages on the Internet (without login verification and access restrictions). The collection and use of image data strictly comply with the terms of the source platform and do not involve personal information, sensitive content or restricted data. The obtained image data is only used for non-commercial scientific research and model training.

8. We have deleted the funding information in the manuscript.
