# Supplementary material for: LPD-YOLOv7-tiny: An enhanced lightweight YOLOv7-tiny model for real-time potato quality detection
Source: PLoS One. 2025 Oct 23;20(10):e0332043. doi: 10.1371/journal.pone.0332043 (PMC12548852; doi:10.1371/journal.pone.0332043)
Supplement: Attachment — Submitted filename: rev.docx [file pone.0332043.s002.docx]

1. In abstract clearly state the *problem*, *method*, *novelty*, and *main results* in four concise sentences.
2. Merge sections 1 and 2 for a smoother transition from problem background to base model.
3. Add a brief model summary table (modules + contributions) before diving into each component.
4. Include a comparison of FLOPs or MACs against original YOLOv7-Tiny. Pls cite this paper as example working with images: Umirzakova, S., Muksimova, S., Mardieva, S., Sultanov Baxtiyarovich, M., & Cho, Y. I. (2024). MIRA-CAP: Memory-Integrated Retrieval-Augmented captioning for State-of-the-Art image and video captioning. *Sensors*, *24*(24), 8013.
5. Add a figure showing latency vs. accuracy trade-off if targeting edge devices.
6. Add comparison with CIOU or EIOU *alone* (ablation against EIOU-only vs. Focal-EIOU).
7. Add confusion matrix per class (Normal/Sprout/Rotten).
