# Supplementary material for: LPD-YOLOv7-tiny: An enhanced lightweight YOLOv7-tiny model for real-time potato quality detection
Source: PLoS One. 2025 Oct 23;20(10):e0332043. doi: 10.1371/journal.pone.0332043 (PMC12548852; doi:10.1371/journal.pone.0332043)
Supplement: Attachment — Submitted filename: Responses to editor (2).docx [file pone.0332043.s004.docx]

## Dear Editor,

We have completed the second round of revisions to the article based on the comments of the reviewers. We are very grateful for the valuable suggestions from the experts and the editor. Your rigorous scientific research attitude enables our research content to be presented better.

Thank you!

Sincerely,

Yongbo Li

College of Intelligent Manufacturing, TaiZhou Polytechnic college

yzdxlyb@163.com
